# LRRK2 phosphorylates novel tau epitopes and promotes tauopathy

**DOI:** 10.1007/s00401-013-1188-4

**Published:** 2013-10-11

**Authors:** Rachel M. Bailey, Jason P. Covy, Heather L. Melrose, Linda Rousseau, Ruth Watkinson, Joshua Knight, Sarah Miles, Matthew J. Farrer, Dennis W. Dickson, Benoit I. Giasson, Jada Lewis

**Affiliations:** 1 Department of Neuroscience, Center for Translational Research in Neurodegenerative Disease, University of Florida, Gainesville, FL 32610 USA; 2 Department of Neuroscience, Mayo Clinic, Jacksonville, FL 32224 USA; 3 Department of Pharmacology, University of Pennsylvania School of Medicine, Philadelphia, PA 19104 USA; 4 Department of Molecular and Cellular Physiology, Stanford University, Stanford, CA 94305 USA; 5 Department of Medical Genetics, University of British Columbia, Vancouver, BC V6T 2B5 Canada; 6 Department of Neuroscience, Center for Translational Research in Neurodegenerative Disease, University of Florida, 1275 Center Drive, BMS Building J-487, PO Box 100159, Gainesville, FL 32610-0244 USA; 7 Department of Neuroscience, Center for Translational Research in Neurodegenerative Disease, University of Florida, 1275 Center Drive, BMS Building J-497, PO Box 100159, Gainesville, FL 32610-0244 USA

## Abstract

Mutations in the gene encoding leucine-rich repeat kinase 2 (LRRK2) are the most frequent cause of familial Parkinson’s disease (PD). The neuropathology of LRRK2-related PD is heterogeneous and can include aberrant tau phosphorylation or neurofibrillary tau pathology. Recently, LRRK2 has been shown to phosphorylate tau in vitro; however, the major epitopes phosphorylated by LRRK2 and the physiological or pathogenic consequences of these modifications in vivo are unknown. Using mass spectrometry, we identified multiple sites on recombinant tau that are phosphorylated by LRRK2 in vitro, including pT149 and pT153, which are phospho-epitopes that to date have been largely unexplored. Importantly, we demonstrate that expression of transgenic LRRK2 in a mouse model of tauopathy increased the aggregation of insoluble tau and its phosphorylation at T149, T153, T205, and S199/S202/T205 epitopes. These findings indicate that tau can be a LRRK2 substrate and that this interaction can enhance salient features of human disease.

**Electronic supplementary material:**

The online version of this article (doi:10.1007/s00401-013-1188-4) contains supplementary material, which is available to authorized users.

## Introduction

Mutations in *leucine-rich repeat kinase 2* (*LRRK2*) are the most frequent cause of autosomal dominant and sporadic cases of Parkinson’s disease (PD) [12, 51, 60, 78]. The functions of LRRK2 are still largely unknown (reviewed in [6]), but aberrations in its kinase activity are thought to lead to pathogenesis [3, 9, 21, 31, 33, 67, 74]. Affected carriers of *LRRK2* mutations are generally clinically indistinguishable from individuals with idiopathic PD and primarily present with Lewy body pathology [3, 19, 26, 61], but neuropathology is pleomorphic and often includes hyperphosphorylated tau protein inclusions [10, 17, 18, 43, 55, 58, 61, 71, 75].

Tau is a soluble protein that binds tubulin to promote microtubule (MT) assembly and support neuronal function (reviewed in [47]). While normal tau function is regulated by phosphorylation, certain phospho-epitopes are considered pathogenic [22] in tauopathies—neurodegenerative diseases that are characterized by the aggregation of hyperphosphorylated tau (reviewed in [68]). Tauopathies include Alzheimer’s disease (AD), progressive supranuclear palsy (PSP), Pick’s disease (PiD), and frontotemporal dementia and parkinsonism linked to chromosome-17 with mutations in the tau gene (FTDP-17*t*), but tau inclusions are often observed in PD brains as well (reviewed in [32]). Furthermore, tau is also present in Lewy bodies in familial and sporadic PD [14, 30]. Although FTDP-17*t* can result from mutations in the gene encoding tau [28, 54, 69], the cause of most tauopathies remains unknown. Given this, identifying tau kinases and determining their involvement in tau pathogenesis are vital to therapeutic targeting of tauopathies.

The appearance of hyperphosphorylated, aggregated tau in the brain of some individuals with *LRRK2* mutations (reviewed in [56]) has led to the suggestion that LRRK2 may be a novel kinase for tau. Several studies, which demonstrated altered tau phosphorylation in transgenic mice expressing mutant LRRK2, support this hypothesis [40, 41, 46]. In addition, recent in vitro and cell culture studies suggest that LRRK2 may phosphorylate tau [35, 71]. If LRRK2 is a novel tau kinase, it is possible that it may phosphorylate novel tau epitopes; however, published studies have focused on a subset of the phospho-epitopes that are frequently associated with human tauopathies. Furthermore, an interaction between LRRK2 and tau has not been directly demonstrated in vivo and it is unclear if such an interaction could influence tau pathologies.

In the current report, we demonstrate that LRRK2 directly phosphorylates tau in vitro and use mass spectrometry (MS) to identify specific tau epitopes that are targets of LRRK2 in vitro. We demonstrate that LRRK2 preferentially phosphorylates tau at T149 and to a lesser extent T153—epitopes that have been largely unexplored by the tau field. We show these epitopes to be hyperphosphorylated in a range of human tauopathies and in individuals with the G2109S LRRK2 mutation using our novel antibodies. Finally, we demonstrate that human wild-type LRRK2 expression in a mouse model of tauopathy enhances tau aggregation and tau hyperphosphorylation—critical features of human tauopathy.

## Materials and methods

Recombinant forms of GST-LRRK2 (970–2,527) were purchased from Invitrogen. Full-length G2019S LRRK2 was cloned into the mammalian expression vector pDEST27, expressed in HEK 293T cells and purified as previously described [8]. The human full-length tau cDNA cloned into the bacterial expression vector pRK172 was kindly provided by Dr. Michel Goedert. Recombinant full-length 0N3R tau and fragments thereof were expressed in *E. coli* BL21 and purified as previously described [27]. Tau mutations (E342V, P301L, P301S, and R406W) were introduced through site directed mutagenesis and verified by DNA sequencing. The mammalian expression plasmid pEF-DEST51 with the full-length wild-type (WT) (with or without a stop codon) or G2019S (with or without a stop codon) LRRK2 cDNAs to generate plasmids expressing full-length untagged LRRK2 (pEF-DEST51-LRRK2, referred to as LRRK2) or full-length LRRK2 with a C-terminal V5-tagged (pEF-DEST51-LRRK2-V5, referred to as LRRK2-V5) were previously described [72]. Synthetic tau peptides TAU-A (KKAKGADGKTKIATPRGAAPPGQK) and TAU-B (REPKKVAVVRTPPKSPSSAKSRL) corresponding to residues 82–105 and 163–185, respectively, in 0N3R tau, as well as threonine to alanine specific mutants were synthesized and purified on reverse phase HPLC by GenScript USA Inc. These peptide sequences correspond to residues 140–163 and 221–243, respectively in 2N4R tau. Recombinant myelin basic protein (MBP) was purchased from Millipore.

### Antibodies

Anti-LRRK2 rabbit polyclonal antibody (1182) was previously described [72]. MJFF-4 (c81-8) was obtained from the Michael J. Fox Foundation. Anti-pT149 and anti-pT153 tau specific antibodies were made as a service by GenScript USA Inc. Briefly, rabbits were immunized with the pT149 peptide (DGKpTKIATPRGAAC) or pT153 peptide (DGKTKIApTPRGAAC), affinity purified with the same peptide, and negatively absorbed against the non-phosphorylated peptide (DGKTKIATPRGAAC). We used polyclonal tau antibodies E1 (specific for amino acids 19–33 of human tau) [11], pT205 (Abcam), pT212 (Anaspec), pS214 (Invitrogen) [70], 17025 anti-tau (provided by Dr. Virginia Lee, University of Pennsylvania, Philadelphia, PA); and monoclonal tau antibodies CP13 (specific for pS202 in tau), PHF1 (specific for pS396/S404 in tau), and MC1 [pre-PHF conformational epitope (7–9 and 326–330)] (provided by Dr. Peter Davies, The Einstein Institute for Medical Research, Manhasset, NY, USA), AT8 (specific for pS199/pS202/pT205 in tau), AT100 (specific for pT212/S214 in tau), and AT270 (specific for pT181 in tau) (Innogenetics, Fisher Scientific). Other monoclonal antibodies used were anti-V5 (Invitrogen) and anti-GAPDH (Biodesign).

### LRRK2 kinase assays

Unless otherwise noted, kinase assays were set up in a total volume of 25 μl with 25 nM recombinant GST–LRRK2 (970–2,527) in 50 mM Tris/HCl (pH 7.5), 0.1 mM EGTA, 10 mM MgCl_2_ and 0.2 mM [γ-^32^P] ATP (4 Ci/mmol) in the presence of 10 μM of tau or MBP substrate, unless otherwise noted. After incubation for 60 min at 30 °C, reactions were terminated by applying to individual 2.5 cm-diameter disks of P-81 phosphocellulose filter paper (Schleicher & Schuell, Keene, NH) that were immediately immersed in 75 mM phosphoric acid. After extensive washing with 75 mM phosphoric acid, P-81 filters were rinsed with acetone and allowed to air dry. Filters were immersed in Cytoscint liquid scintillation cocktail (Fisher Scientific) and ^32^P radioactivity on each filter was measured by liquid scintillation using an LS6500 counter (Beckman Coulter). *K*_m_ and *V*_max_ parameters were calculated using Graph-Pad Prism v5.02 (GraphPad Software).

For phosphorylation analysis of recombinant tau proteins with LRRK2, reactions were stopped with the addition of SDS-sample buffer and heating to 100 °C for 5 min. Samples were resolved onto SDS-polyacrylamide gels, and incorporation of phosphate was determined by autoradiography and/or immunoblotting with phospho-specific antibodies.

### Mass spectrometry

As described above, 10 μM of 0N3R tau was subjected to overnight in vitro phosphorylation by either G2019S LRRK2 or the kinase dead (KD) LRRK2 mutant D1994K. Samples were resolved onto SDS-polyacrylamide gels, and then excised and washed in 50 % acetonitrile and water [5]. To maximize sequence coverage, three equivalently loaded and excised gel bands of 0N3R tau were separately digested with trypsin, chymotrypsin or GluC. Mass spectrometry (MS) analysis of modified 0N3R tau was performed as a service by the Harvard Mass Spectrometry and Proteomics Resource Laboratory (Cambridge, MA, USA). Peptide sequence analysis of each digestion mixture was performed by microcapillary reversed-phase high-performance liquid chromatography coupled with nanoelectrospray tandem mass spectrometry (μLC–MS/MS) on an LTQ-Orbitrap Velos mass spectrometer (ThermoFisher Scientific, San Jose, CA, USA). The Orbitrap repetitively surveyed an *m*/*z* range from 395 to 1,600, while data-dependent MS/MS spectra on the twenty most abundant ions in each survey scan were acquired in the linear ion trap. MS/MS spectra were acquired with relative collision energy of 30 %, 2.5-Da isolation width, and recurring ions dynamically excluded for 60 s. Preliminary sequencing of peptides was facilitated with the SEQUEST algorithm [15] with a 30 ppm mass tolerance against the human subset of the Uniprot Knowledgebase supplemented with a database of common laboratory contaminants, concatenated to a reverse decoy database. Using a custom version of Proteomics Browser Suite (PBS v.2.7, ThermoFisher Scientific, San Jose, CA, USA) peptide-spectrum matches (PSMs) were accepted with mass error <2.5 ppm and score thresholds to attain an estimated false discovery rate of ~1 %. Data-sets for all digest results were combined in silico, culled of minor contaminant PSMs, and re-searched with SEQUEST algorithm against the 0N3R tau sequence without taking into account enzyme specificity and with differential modifications of phosphorylated tyrosine, serine, and threonine residues. The discovery of phosphopeptides and subsequent manual confirmation of their MS/MS spectra were facilitated using in-house versions of programs MuQuest, GraphMod, and FuzzyIons (PBS, ThermoFisher Scientific).

### Enzyme-linked immuno sorbent assay (ELISA) to assess antibody specificity

Recombinant C-terminal fragment of human 3R tau [C′ Tau] corresponding to amino acids 244–441 minus amino acids 275–305 that would be present in 2N4R tau human tau was phosphorylated with recombinant glycogen synthase kinase 3 beta (GSK-3β) (New England BioLabs Inc, Ipswich, MA, USA) (20 mM Tris, pH 7.5, 10 mM MgCl_2_, 5 mM DTT, 200 μM ATP, 1 mg/ml tau and 20,000 units enzyme in 50 μl) for 1 h at 30 °C. Controls included similar reactions without the kinase. 100 ng of each synthetic peptide or 500 ng of tau recombinant protein diluted in 100 μl of water was absorbed per well of 96-well EIA/RIA Corning plates (Corning, NY, USA). The plates were extensively washed with PBS and blocked with PBS/5 % fetal bovine serum (FBS). Primary antibodies as indicated were diluted in PBS/5 % FBS and incubated on the plates. After extensive washes with PBS, plates were incubated with either anti-mouse antibody conjugated to HRP or anti-rabbit antibody conjugated to HRP diluted in PBS/5 % FBS. Plates were again extensively washed with PBS and reactions were carried out with 3,3′,5,5′-tetramethylbenzidine (TMS) reagents (KPL, Gaithersburg, NY, USA). The reactions were terminated by adding 0.2 M HCl and optical density was measured at OD_450_ using a Multiskan Plus plate reader (ThermoFisher). Experiments were performed in quadruplicate.

### Cell culture

HEK 293T cells were cultured in Dulbecco’s modified medium with high glucose (4.5 g/l) supplemented with 10 % FBS, 100 U/ml penicillin, 100 U/ml streptomycin, and 2 mM l-glutamine. Cells were plated onto 6-well plates and transfected at approximately 30 % confluence using Lipofectamine 2000 according to the manufacturer’s protocol. Cells were maintained for 48 h after transfection. Cells were harvested and lysed in 3 % SDS/50 mM Tris–HCl, pH 6.8 and heated to 100 °C for 5 min. Protein concentration was determined using BCA protein assay reagent and BSA as the standard (Pierce, Fisher Scientific). Experiments were performed in duplicate.

### Mice

Mice were housed and treated in accordance with the NIH Guide for the Care and Use of Laboratory Animals. All animal procedures were approved and conducted in accordance with the Mayo Clinic Institutional Animal Care and Use committee and the University of Florida Institutional Animal Care and Use committee. Mice were maintained in a pathogen-free facility on a 12 h light/dark cycle with water and food provided ad libitum.

The parental Tau_P301L_ responder line and parental tTA activator line were generated and maintained on an FVB and 129/S6 background, respectively, as previously described [65]. Bigenic rTg4510 mice have forebrain-focused expression of P301L transgenic tau. The parental bacterial artificial chromosome (BAC)-LRRK2 mice, maintained on an FVB background, contain the entire human *LRRK2* gene including regulatory sequences and LRRK2 expression is driven by the human *LRRK2* promoter, as previously described [46]. Mice from the Tau_P301L_ responder line were crossed with mice from the BAC-LRRK2 mouse line for one generation to obtain LRRK2.Tau_P301L_ responder mice on an FVB background. LRRK2.Tau_P301L_ responder mice were then crossed with mice from the tTA activator line to obtain the resultant F1 LRRK2/Tau_P301L_ mice on a 50 % FVB, 50 % 129S background, the same as the original rTg4510 mouse model [65]. All mice in this study were harvested at 5.5 months of age when mature cortical tangles and hippocampal neurodegeneration are detectable in Tau_P301L_ only animals. We harvested 10 mice per group, half male and half female, for the four genotypes of interest (non-transgenic, LRRK2, Tau_P301L_, and LRRK2/Tau_P301L_). One male LRRK2/Tau_P301L_ mouse did not overexpress LRRK2 (as determined by western blotting) and was excluded from all studies. The sarkosyl-preparation (see protocol below) of one female Tau_P301L_ mouse had extremely high tau aggregation via western blotting and was identified by Grubb’s analysis to be an outlier. As this may have been a tissue preparation error, all western blot data from this mouse were excluded from the final results.

### Tissue harvest and preparation

All mice were euthanized by cervical dislocation to maintain the brain biochemistry by avoiding anesthesia-induced tau changes. Brains were quickly removed, cut down the midline, and one brain half was drop fixed in 10 % formalin for 24 h for immunohistochemical analysis and the other brain half was immediately homogenized to preserve the LRRK2 protein. Brains were homogenized in 6 volumes of homogenate buffer [50 mM Tris–HCl, pH 8.0, 274 mM NaCl, 5 mM KCl, 1 % protease inhibitor mixture (Sigma), 1 % phosphatase inhibitor cocktails I and II (Sigma), and 1 mM phenylmethylsulfonyl fluoride (PMSF)]. For LRRK2 analysis, homogenates were centrifuged at 150,000×*g* for 15 min at 4 °C. The supernatants were collected and protein concentration was determined using BCA protein assay reagent and BSA as the standard. For tau analysis, the brain homogenate was diluted to 10 volumes using Tris-buffered saline and subjected to sarkosyl fractionation as previously described [65]. Specifically, 200 μl of 10× homogenate was centrifuged at 150,000×*g* for 15 min at 4 °C and the supernatant, which contains soluble tau species, was collected and protein concentration was determined as described above. Pellets were homogenized in 3 volumes (600 μl) of Buffer B [10 mM Tris–HCl (pH 7.4), 0.8 M NaCl, 10 % Sucrose, 1 mM EGTA and 1 mM PMSF] and centrifuged at 150,000×*g* for 15 min at 4 °C. The supernatants were collected and incubated with 1 % sarkosyl (Sigma) for 1 h at 37 °C, followed by centrifugation at 150,000×*g* for 30 min at 4 °C to obtain a sarkosyl-soluble supernatant and sarkosyl-insoluble pellet. The sarkosyl-insoluble pellet, which contains the biochemical equivalent of neurofibrillary tangles, was re-suspended in 20 μl TE buffer [10 mM Tris–HCl (pH 8.0), 1 mM EDTA].

### Western blot analysis

For in vitro and cell culture experiments, equal amounts of protein samples were loaded and resolved by SDS-PAGE, followed by electrophoretic transfer onto nitrocellulose membranes. Membranes were blocked in Tris-buffered saline (TBS) with 5 % non-fat milk powder, and incubated overnight with 1182 LRRK2-specific antibody [72], anti-V5 antibody (Invitrogen), or anti-tau antibody (17025) in TBS/5 % non-fat milk powder. Membranes were also incubated overnight with pT149 or pT153 tau specific antibodies in TBS/5 % BSA. Each incubation was followed by goat anti-mouse conjugated horseradish peroxidase (HRP) (Amersham Biosciences) or goat anti-rabbit HRP (Cell Signaling Technology), and immunoreactivity was detected using chemiluminescent reagent (NEN) followed by exposure on X-ray film.

For analysis of LRRK2 protein in mouse brain tissue, 50 μg of protein was diluted in NuPAGE LDS-sample buffer with NuPAGE reducing agent (Invitrogen), boiled for 3 min at 95 °C, loaded onto 26-well 3–8 % Tris–Acetate gels (Invitrogen), separated by SDS-PAGE and transferred in NuPAGE transfer buffer (Invitrogen) to PVDF membranes (Millipore, Fisher Scientific). Membranes were blocked for 1 h at room temperature in 5 % non-fat milk powder in TBS with 0.1 % Triton X-100 (TBS-T) and then incubated overnight in primary antibody diluted in 5 % non-fat milk powder in TBS-T at 4 °C. For tau protein analysis from mouse brain, 5 μg of the soluble fraction or 3 μl of the sarkosyl-insoluble fraction was diluted in Novex Tris–glycine SDS-sample buffer (Invitrogen) with β-mercaptoethanol, heat denatured at 95 °C for 5 min, loaded onto 26-well 10 % Tris–glycine gels (Invitrogen), separated by SDS-PAGE and transferred in CAPS transfer buffer (Sigma) to PVDF membranes. Membranes were blocked in TBS-T with 5 % non-fat milk powder and incubated overnight with antibody in TBS-T/5 % non-fat milk powder, except for pT149 and pT153 tau antibodies, which were in TBS-T/5 % BSA. All membranes were washed 3 times in TBS-T, incubated with either goat anti-mouse HRP or goat anti-rabbit HRP secondary antibodies (Jackson ImmunoResearch) for 1 h at room temperature and washed again 3 times in TBS-T. Membranes were developed using Western Lightning Plus (Perkin Elmer) and imaged using a FluorChem E System (ProteinSimple). The relative levels of immunoreactivity were determined by densitometry using the software AlphaView SA (ProteinSimple).

### Immunohistochemistry

Fixed mouse brains were paraffin embedded and cut into 5 μm sagittal sections. Hematoxylin and eosin (H&E) staining was performed on at least two brain sections from each mouse to align all brains to approximately 1.3 mm lateral to the midline using a mouse brain atlas [52]. Immunohistochemistry with the Dako Universal Autostainer with DAKO Envision + HRP system (Dako) was performed with the following antibodies: pT149 tau, pT153 tau, AT8, AT270, CP13, and MC1. Stained slides were digitally scanned using a ScanScope XT scanner and were analyzed using ImageScope version 11.2.0.780 software (Aperio). Positive pixel count algorithms were created to measure the staining density of the secondary antibody, specifically chromogen 3,3′ diaminobenzidine. The cortex of each animal was traced and analyzed using these algorithms and the burden was expressed as a percentage of immunostained pixels to total area. Sections that had tears or other artifacts were not included in the analysis.

### Human tissue

Paraffin embedded sections were processed for immunohistochemistry as above for mice from 3R + 4R tauopathy (5 AD—average age: 90 ± 6 years; 4 women; median Braak NFT Stage: VI; 2 with concurrent diffuse cortical Lewy bodies), 4R tauopathy (4 PSP and 1 CBD—average age: 77 ± 7 years; 4 women; median Braak NFT Stage: III), 3R tauopathy (2 PiD—average age: 68 ± 6 years; 1 woman; median Braak NFT Stage: III), and mutant LRRK2 carriers (4 G2019S—average age: 76 ± 5 years; 2 women; Braak NFT Stage: 0–V). Sections were counterstained with hematoxylin.

### Statistical analysis

Grubb’s analysis was used to identify outliers in the western blot analysis of sarkosyl-insoluble tau. Data are presented as the mean ± standard error mean unless otherwise noted. Analysis of two groups was performed with an unpaired, two-tailed Student’s *t* test while analysis of multiple groups was analyzed by one-way ANOVA and post hoc Bonferroni multiple comparisons test (*α* = 0.05). Western blot and immunohistochemical data from male and female Tau_P301L_ and LRRK2/Tau_P301L_ mice were analyzed by two-way ANOVA with genotype and sex as independent variables. All analyses were performed using GraphPad Prism version 6.00 software (GraphPad Software) and in all cases, *P* ≤ 0.05 was considered to be statistically significant.

## Results

### Wild-type and mutant LRRK2 phosphorylates multiple tau isoforms in vitro

**Fig. 1:**
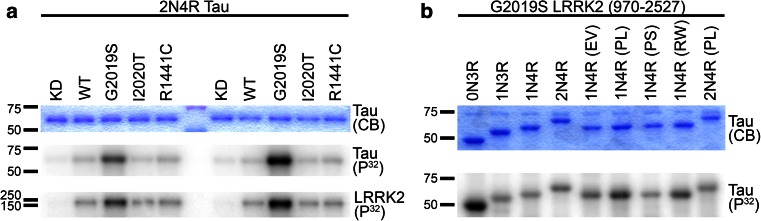
Wild-type and mutant LRRK2 phosphorylates tau in vitro. **a** Wild-type (WT) and mutant LRRK2 proteins directly phosphorylate 2N4R tau in vitro. Recombinant human tau (2N4R) is phosphorylated by recombinant WT, G2019S, I2020T, and R1441C human GST-LRRK2 (970–2,527), but not the kinase dead (KD) D1994K mutant as shown in duplicate by autoradiography (P^32^). Kinase activity of each LRRK2 construct was validated by LRRK2 auto-phosphorylation. **b** G2019S LRRK2 directly phosphorylates multiple tau isoforms with and without FTDP-17*t* mutations. Recombinant GST-G2019S LRRK2 (970–2,527) phosphorylates tau, regardless of tau isoform or mutation as shown by autoradiography (P^32^). In both **a** and **b**, Coomassie Blue (CB) shows equal loading of tau. Tau mutation abbreviations: *EV* E342V, *PL* P301L, *PS* P301S, *RW* R406W

To determine if LRRK2 directly phosphorylates tau, recombinant tau (2N4R) was incubated with multiple forms of recombinant GST-LRRK2 (970–2,527) and incorporation of γ-[^32^P] ATP was determined by autoradiography. Both wild-type (WT) and mutant (G2019S, I2020T and R1441C) LRRK2 phosphorylated recombinant tau in vitro (Fig. 1a). Although we analyzed three different LRRK2 mutants, each of which has been implicated in familial PD with neurofibrillary tangle pathology, G2019S LRRK2 yielded the highest level of tau phosphorylation. Further, tau phosphorylation was largely ablated when using the LRRK2 kinase dead/impaired (KD) D1994K mutant (Fig. 1a), indicating that LRRK2 directly phosphorylated tau in vitro. We then examined the ability of mutant LRRK2 to phosphorylate different tau isoforms that contain alternatively spliced N-terminal (0, 1 or 2 N) and C-terminal MT-binding domains (3R or 4R). Recombinant GST-G2019S LRRK2 (970–2,527) phosphorylated all forms of tau regardless of isoform or the presence of FTDP-17*t*-associated mutations (E342V, P301L, P301S and R406W) (Fig. 1b). These findings were confirmed using full-length G2019S LRRK2 (Supplemental Fig. 1).

### Identification and characterization of tau residues phosphorylated by LRRK2 in vitro

**Fig. 2:**
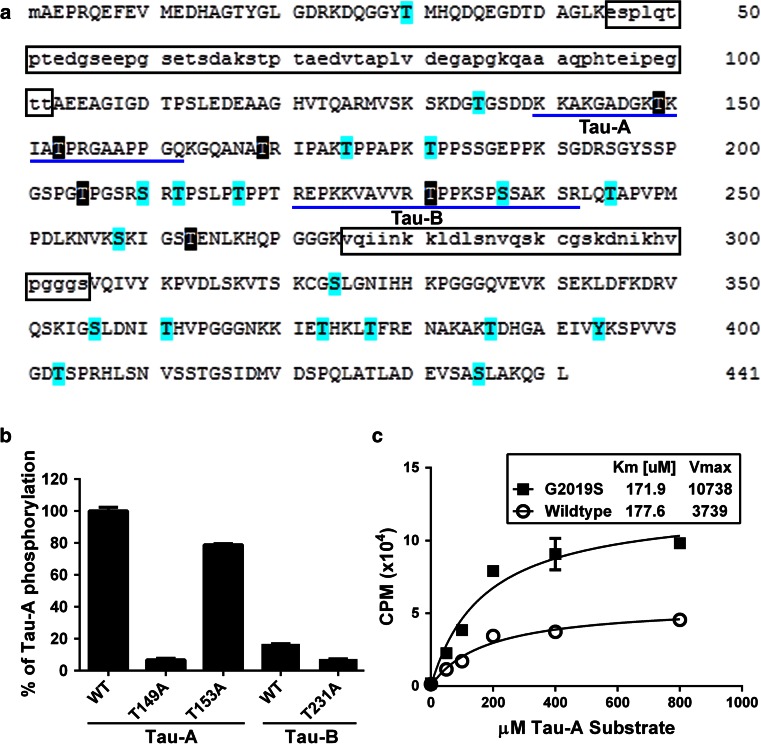
LRRK2 phosphorylates multiple tau epitopes in vitro. **a** Highlighted amino acids indicate residues of 0N3R tau phosphorylated by GST-G2019S LRRK2 (970–2,527), but not by kinase dead GST-LRRK2 (970–2,527) as identified as putative sites by mass spectrometry. We obtained coverage of ~99 % of the 0N3R construct as indicated by capital letters. Major phosphorylation sites are highlighted in black. The entire amino acid sequence for the longest human tau isoform, 2N4R, is denoted and the tau domains, encoded by the alternatively spliced exons 2, 3, and 10 that were not included in the 0N3R tau construct are boxed. **b** We compared GST-G2019S LRRK2 (970–2,527) phosphorylation using synthetic peptides (Tau-A and Tau-B) as indicated by blue underlines in **a**. The degree of G2019S LRRK2-directed phosphorylation of WT Tau-A (T149, T153) and Tau-B (T231) peptides was compared to Tau-A and Tau-B with individual alanine mutants (T149A, T153A, T231A), which blocked phosphorylation at each residue, to determine the relative substrate potential of each epitope. **c** Kinetics of GST-WT and -G2019S LRRK2 (970–2,527) phosphorylation of Tau-A peptide. The concentration of Tau-A substrate was varied as indicated while the concentration of ATP was constant at 200 μM

To identify the tau residues that are phosphorylated by LRRK2 in vitro, we performed MS analyses of tau (0N3R) phosphorylated with GST-G2019S LRRK2 (970–2,527). Using the shortest tau isoform with the highest kinase active LRRK2, we had the greatest likelihood of complete coverage. We obtained >99 % sequence coverage from the 0N3R isoform and identified multiple tau residues phosphorylated by G2019S LRRK2 (Fig. 2a), with T149, T153, T169, T205, T263 and T231 identified as the major phosphorylation sites by LRRK2 (Supplemental Table 1). These modifications were not present in identical MS analysis of 0N3R tau incubated with KD LRRK2. To parse the sites that were preferentially phosphorylated by LRRK2, we incubated various forms of recombinant LRRK2 with synthetic peptides that spanned short regions of tau containing 1–3 potential tau phospho-epitopes (Supplemental Table 2) and quantified the resultant phosphorylation. G2019S-LRRK2 showed the greatest phosphorylation of the Tau-A (KKAKGADGKTKIATPRGAAPPGQK) and Tau-B (REPKKVAVVRTPPKSPSSAKSRL) peptides, with the WT Tau-B peptide phosphorylated 83 ± 1.7 % (*P* < 0.001) less than the WT Tau-A peptide (Fig. 2b) as assessed by one-way ANOVA and post hoc Bonferroni multiple comparisons test. The other peptides demonstrated minimal phosphorylation. These data were consistent with the MS data, which showed that pT149 and pT153 peptides were among the most abundant phosphorylated products resulting from LRRK2-mediated tau phosphorylation. Alanine substitution to block site-specific LRRK2 phosphorylation of the Tau-A peptide reduced phosphorylation by 93 ± 1.2 % (T149A; *P* < 0.0001) and 21 ± 1.2 % (T153A; *P* < 0.001) compared to WT Tau-A (Fig. 2b), indicating that tau T149 and T153 were primary and secondary targets, respectively, of LRRK2-directed phosphorylation. We then assessed the kinetic features of WT and G2019S LRRK2 phosphorylation of Tau-A (see “Materials and methods”) where the concentration of Tau-A was varied as indicated while the concentration of ATP was constant at 200 μM. The *K*_m_ of WT and G2019S LRRK2 for the Tau-A peptide was determined to be ~170 μM, which is similar to that of LRRKtide, the most robust and commonly used LRRK2 substrate in kinetic studies [1, 9, 31, 48]. Furthermore, like LRRKtide, the *V*_max_ of tau phosphorylation by G2019S LRRK2 was ~threefold greater than that of WT LRRK2 (Fig. 2c) [9].

### Tau is phosphorylated at T149 and T153 in G2019S carriers and in a range of human tauopathies

**Fig. 3:**
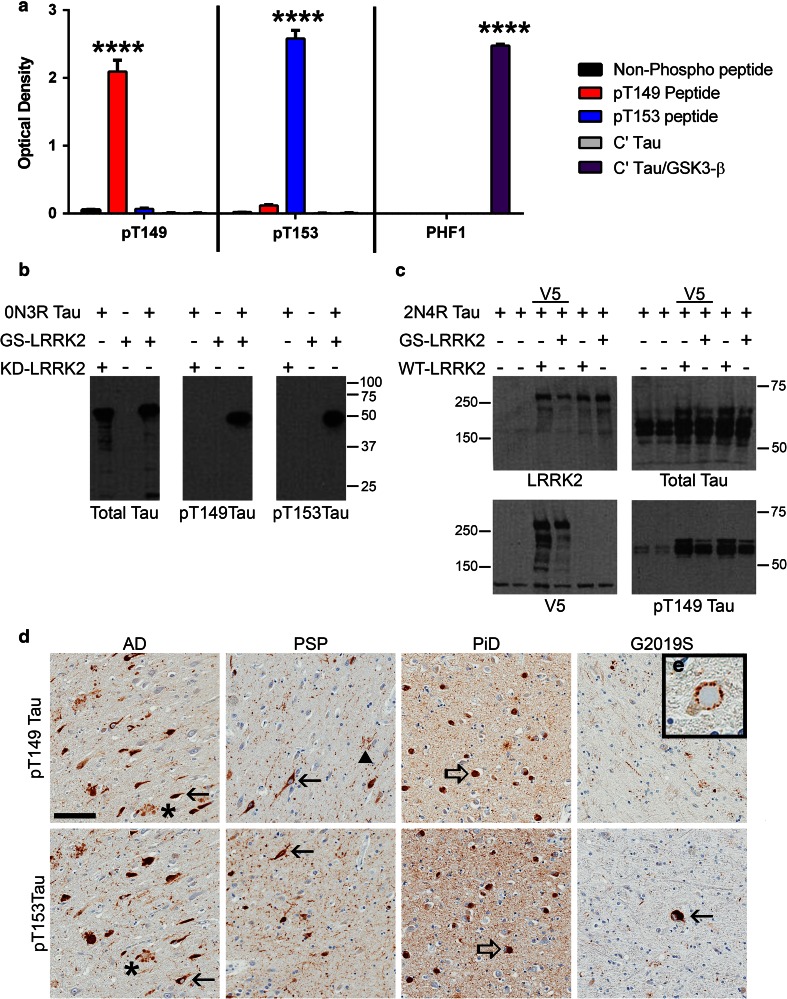
Novel pT149 and pT153 tau antibodies recognize human tau pathology. **a** ELISA of phosphorylated and non-phosphorylated tau peptides with antibodies to pT149, pT153 and the PHF1 antibody, as a well-characterized control. The pT149 antibody reacted strongly with tau peptides phosphorylated at T149 while the pT153 antibody had the greatest interaction with tau phosphorylated at T153. Neither pT149 nor pT153 tau antibodies reacted with non-phosphorylated tau nor with the C-terminal half of 3R tau (C′ Tau) phosphorylated by GSK-3β. The pT153 antibody had low cross reactivity with tau phosphorylated at T149 as compared to non-phosphorylated C′ Tau, but not the other control peptides. ****P < 0.0001 [two-way ANOVA (antibody × peptide) with post hoc Bonferroni multiple comparisons test] (**b**) 0N3R tau was subjected to in vitro phosphorylation by either GST-G2019S (GS) or kinase dead (KD) LRRK2 (970–2,527) and analyzed via western blotting with 17025 (total tau), pT149 and pT153 antibodies. pT149 and pT153 antibodies recognize tau phosphorylated in vitro by G2019S LRRK2, but not tau incubated with the KD LRRK2. **c** HEK 293T cells were co-transfected with expression plasmids for 2N4R tau and pcDNA3.1, WT LRRK2-V5, GS LRRK2-V5, WT LRRK2 or GS LRRK2 and cell lysates were analyzed via western blot with LRRK2 (1182), V5, 17025 (total tau) and pT149 tau antibodies. Our novel pT149 antibody specifically detects tau phosphorylated by full-length WT and GS LRRK2 in cell culture. **d** In human tauopathies, pT149 (*top panels*) and pT153 (*bottom panels*) tau antibodies recognized neurofibrillary tangles (*filled arrows*) and neuritic tau pathology (*asterisk*) associated with plaques in Alzheimer’s disease (AD, *left panels*), neurofibrillary tangles (*filled arrows*) and tufted astrocytes (*arrowhead*) in progressive supranuclear palsy (PSP, *middle-left panels*), Pick bodies (*open arrows*) in Pick’s disease (PiD, *middle-right panels*), and recognized neurofibrillary tangles (*filled arrow*) in G2019S LRRK2 patients (G2019S, *right panels*). **e** The pT149 antibody stains tau pathology surrounding a Lewy body in the midbrain. *Scale bar*, 100 μm for panel **d**. Panel (**e**) is 50 μm × 50 μm

To assess phosphorylation of tau T149 or T153, we created antibodies specific for each phospho-epitope (see “Materials and methods”). ELISA analysis of pT149 and pT153 tau antibodies was used to establish the phospho-specificity of each antibody. Results showed that both antibodies are highly specific for tau peptides phosphorylated at their respective epitopes with minimal reactivity to non-phosphorylated tau peptide, non-phosphorylated C-terminal tau [C′ Tau] and C′ Tau phosphorylated by GSK-3β (Fig. 3a). In addition, pT149 antibody had minimal cross reactivity to tau peptide phosphorylated at T153 and the pT153 antibody had low cross reactivity to tau peptide phosphorylated at T149, but none when compared to the other control peptides. Both pT149 and pT153 antibodies recognized tau that had been incubated with GST-G2019S LRRK2 (970–2,527), but not with GST-KD LRRK2 (970–2,527) (Fig. 3b). Further, HEK 293T cells were co-transfected with expression plasmids for 2N4R tau and pcDNA3.1, untagged full-length WT or G2019S LRRK2, or V5-tagged full-length WT or G2019S LRRK2. Immunoblot analysis of cell lysates with total tau and pT149 tau antibodies confirmed that both tagged and untagged full-length WT and G2019S LRRK2 increased phosphorylation of tau at T149 in vivo by 8.4 ± 0.8-fold more (WT; *P* < 0.0001) and 7.5 ± 0.4-fold more (G2019S; *P* < 0.0001) than vector transfected control cells when normalized to total tau (Fig. 3c).

To test the pathological relevance of pT149 and pT153, we performed immunohistochemistry on brain tissue from different human tauopathies using our pT149 and pT153 tau antibodies. Both antibodies recognized neurofibrillary pathology in AD (Fig. 3d, left panels), including neurofibrillary tangles, neuropil threads and dystrophic neurites in senile plaques. Further, both antibodies stained neurofibrillary tangles and tufted astrocytes (Fig. 3d, middle-left panels) in PSP, as well as Pick bodies in PiD (Fig. 3d, middle-right panels). Moreover, the pT149 and pT153 antibodies stained pathological tau in patients with the G2019S LRRK2 mutation (Fig. 3d, right panels). In addition to the tangle pathology previously reported for G2019S mutant carriers, our antibodies identified tau surrounding Lewy bodies in the brainstem (Fig. 3e). These results indicate that in addition to G2019S-LRRK2 carriers, tau hyperphosphorylation at T149 and T153 occurs in a range of human tauopathies, including so-called 4R-tauopathies, 3R-tauopathies and 3R + 4R tauopathies, and that phosphorylation of these epitopes is linked to pathology, since no staining was detected in unaffected brains or unaffected brain regions in tauopathies (data not shown).

### LRRK2 does not alter levels or phosphorylation of soluble tau in LRRK2/Tau_P301L_ mice

To determine, if LRRK2 could modify the development of tauopathy in vivo, we crossed human WT LRRK2 BAC mice that do not have an overt phenotype or pathological abnormalities (including evidence of tau pathology) [46], with rTg4510 mice that express human mutant (P301L) 0N4R tau [65] to generate mice that expressed both transgenes (LRRK2/Tau_P301L_) (see breeding scheme in Supplemental Fig. 2). LRRK2 BAC mice showed the highest expression of transgenic LRRK2 in the hippocampus as well as high expression in the cortex [46]. These regions overlap well with the forebrain-focused transgenic tau expression that is conditionally driven by a CAMKIIα-tTA transgene in the rTg4510 mice [65], reducing the likelihood that an interaction between the proteins would be missed due to disparate expression profiles. The forebrain of 5.5-month-old rTg4510 mice, hereafter termed Tau_P301L_, has been shown previously to have insoluble hyperphosphorylated tau, neurofibrillary tangles, and neuronal loss [59, 65]. We, therefore, examined LRRK2/Tau_P301L_ mice at 5.5 months of age for alterations in this well-characterized pathology.

**Fig. 4:**
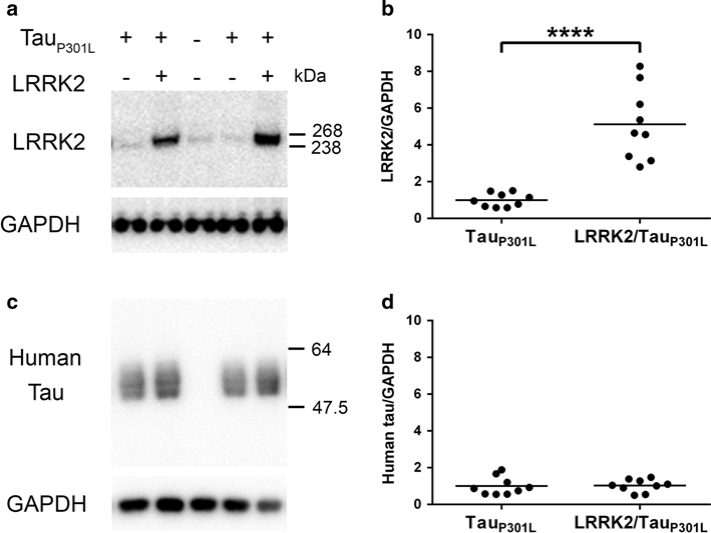
Tau expression in Tau_P301L_ transgenic mice is unaffected by addition of transgenic LRRK2 expression in Tau_P301L_ mice. The steady state amounts of LRRK2, human tau and GAPDH (loading control) were estimated by western blot analyses of the soluble fractions of whole brain lysates from Tau_P301L_ and LRRK2/Tau_P301L_ mice. **a** Representative western blot of LRRK2 and GAPDH with a non-transgenic littermate shown as a negative control. **b** Densitometric quantification of LRRK2 levels normalized to GAPDH. **c** Representative western blot of soluble human tau and GAPDH. **d** Densitometric quantification of human tau levels normalized to GAPDH. *Each dot* represents an individual mouse with the mean indicated by the *black line*, *n* = 9 per cohort. ****P < 0.0001 [two-way ANOVA (genotype × sex): main effect of genotype indicated]

Initially, we confirmed that all LRRK2/Tau_P301L_ experimental mice expressed the LRRK2 (Fig. 4a, b) and tau (Fig. 4c, d) transgenes. Soluble tau fractions typically contain normal tau species that run at ~55 kDa which can be used to determine equal levels of expression across mice. Human LRRK2 expression in LRRK2/Tau_P301L_ mice did not affect human tau levels in the normal (soluble) fraction (Fig. 4c, d) when compared to Tau_P301L_ mice alone, ensuring that any effects on tauopathy in the LRRK2/Tau_P301L_ mice would not be due to expression artifact. With a panel of phospho-specific tau antibodies, we then analyzed soluble tau via western blotting for phosphorylation at sites that we confirmed to be directly phosphorylated by LRRK2 in vitro (T149 and T153) and/or identified by our initial MS results (T181, T212 and T205). We also included epitopes that are associated with human disease and were not identified by our MS analysis (S202, S214, and S396/S404). We did not detect any changes in the phosphorylation of the ~55 kDa soluble tau in LRRK2/Tau_P301L_ mice when compared to the Tau_P301L_ mice with any antibody utilized (Supplemental Fig. 3). We and others have previously reported that species of higher molecular weight tau can be observed in the soluble fraction, potentially representing some species of aggregated tau [39, 63, 64]. Interestingly, our pT153 antibody recognized a ~64 kDa band in the soluble fraction of both Tau_P301L_ and LRRK2/Tau_P301L_ mice (Supplemental Fig. 3a). Comparison of this band between both cohorts (*P* = 0.17) failed, however, to reach our 0.05 threshold for significance.

### Accumulation and phosphorylation of insoluble tau is increased in LRRK2/Tau_P301L_ mice

**Fig. 5:**
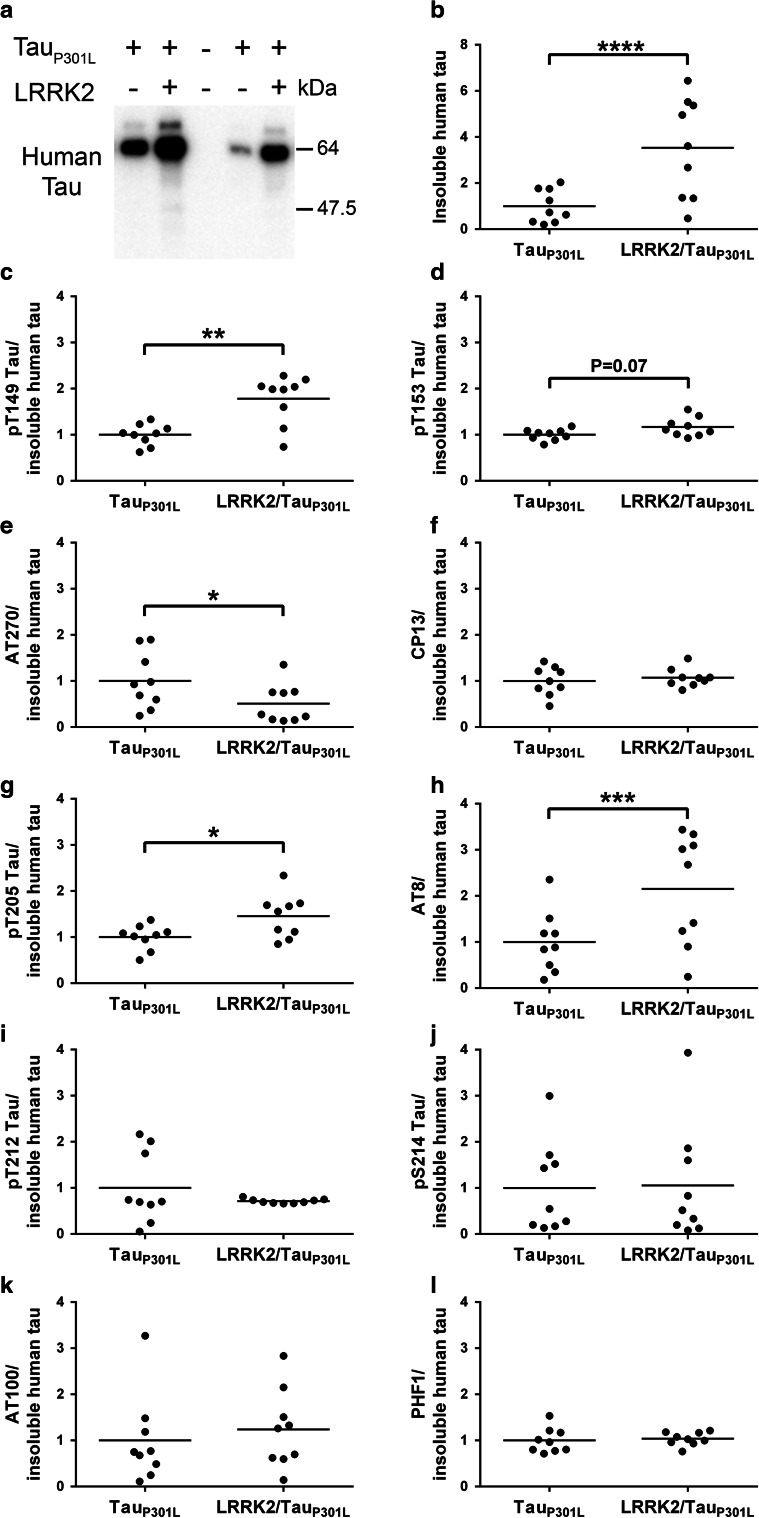
LRRK2/Tau_P301L_ mice have elevated insoluble, hyperphosphorylated tau compared to Tau_P301L_. Western blot analyses of the sarkosyl-insoluble fractions of whole brain lysates from Tau_P301L_ only and LRRK2/Tau_P301L_ mice. **a** Representative immunoblot probed with an antibody (E1) that recognizes human tau, regardless of phosphorylation state, shows significantly more aggregated (insoluble) human tau in LRRK2/Tau_P301L_ compared to Tau_P301L_ only mice as measured by (**b**) densitometry. Note the ~64 kDa species of tau (**a**) that we have previously shown to be a highly aggregated, hyperphosphorylated form of 0N4R tau [64]. **c**–**l** Levels of insoluble tau phosphorylated at T149 (**c**), T153 (**d**), T181 (AT270) (**e**), S202 (CP13) (**f**), T205 (**g**), S199/S202/T205 (AT8) (**h**), T212 (**i**), S214 (**j**), T212/S214 (AT100) (**k**), and S396/S404 (PHF1) (**l**), as measured by densitometry (Supplemental Fig. 6), were adjusted by levels of human tau in the insoluble fraction (**a-b**) to distinguish specific phospho-epitopes that are enriched in the insoluble fraction. Phosphorylation of insoluble tau is increased at T149, T205 and S199/S202/T205 and decreased at T181 when normalized to the amount of tau in the sarkosyl-insoluble fraction. *Each dot* represents an individual mouse with the mean indicated by the *black line*, *n* = 9 per cohort. *P ≤ 0.05, **P < 0.01, ***P < 0.001, ****P < 0.0001 [two-way ANOVA (genotype × sex): main effect of genotype indicated]. Summary of two-way ANOVA found in Table 1

An important feature of human tauopathy and a subset of mutant LRRK2 carriers is the aggregation of tau. In tauopathies, tau aggregation is associated with the shift of tau into the biochemically abnormal, sarkosyl-insoluble fraction. We, therefore, examined the sarkosyl-insoluble fractions of brains from LRRK2/Tau_P301L_ mice by western blot analysis to determine if LRRK2 enhances tau aggregation compared to Tau_P301L_ mice. Using the phospho-independent, human tau antibody, E1, we found that insoluble, aggregated tau levels were ~3.5 times higher in LRRK2/Tau_P301L_ compared to Tau_P301L_ mice (Fig. 5a, b). Insoluble tau from both LRRK2/Tau_P301L_ and Tau_P301L_ mice migrated primarily as a ~64 kDa band—a species that we have previously shown to correlate with the presence of NFTs in tau transgenic mice [39]. Using a two-way ANOVA (genotype × sex), we then determined that the levels of sarkosyl-insoluble human tau in Tau_P301L_ and LRRK2/Tau_P301L_ mice were affected by both genotype [*F*(1, 14) = 34, *P* < 0.0001] and sex [*F*(1, 14) = 34, *P* < 0.0001], and that there was also a significant interaction between both factors [*F*(1, 14) = 14, *P* < 0.01]. To help interpret these findings, we analyzed females and males separately and found that female LRRK2/Tau_P301L_ mice had approximately 3.6 times more insoluble tau compared to female Tau_P301L_ mice (*P* < 0.001) (Supplemental Fig. 4a), and that male LRRK2/Tau_P301L_ mice had 2.8 times more insoluble tau compare to male Tau_P301L_ mice (*P* < 0.05) (Supplemental Fig. 4b).

We then analyzed the sarkosyl-insoluble fraction of Tau_P301L_ and LRRK2/Tau_P301L_ mouse brains using our panel of phospho-tau antibodies and found that LRRK2 increased phosphorylation of insoluble tau at all epitopes that were examined (Supplemental Figs. 5 and 6). To determine if the elevated levels of phosphorylated tau were simply driven by the overall increase of aggregated tau in the insoluble fraction, we normalized the densitometry of each phospho-tau antibody by the densitometry of sarkosyl-insoluble human tau for each mouse. When normalized for the amount of insoluble tau in the sarkosyl-insoluble fraction, analysis by two-way ANOVA (genotype × sex) revealed a main genotype effect with increased phosphorylation of T149, T205, and S199/S202/T205 (Fig. 5c, g, h; Table 1) and decreased phosphorylation of T181 in LRRK2/Tau_P301L_ mice compared to Tau_P301L_ mice (Fig. 5e; Table 1). Phosphorylation of T153 in LRRK2/Tau_P301L_ mice compared to Tau_P301L_ mice (*P* = 0.07) did not reach our significance threshold (Fig. 5d; Table 1) and phosphorylation of S202, T212, S214, T212/S214 and S396/S404 was unchanged by co-expression of LRRK2 in Tau_P301L_ mice (S202: *P* = 0.62; T212: *P* = 0.32; S214: *P* = 0.90; T212/S214: *P* = 0.63; and S396/S404: *P* = 0.40) (Fig. 5f, i–l; Table 1). In addition, this analysis revealed a main sex effect for pT181 (AT270), S199/S202/T205 (AT8) and S396/S404 (PHF1), but not the other epitopes examined (see statistical summary in Table 1).

**Table 1:** Summary of sarkosyl-insoluble and immunohistochemical analysis of tau measured from female and male Tau_P301L_ and LRRK2/Tau_P301L_ mice

Stain	Genotype		Sex		Interaction	
	*F*(DFn, DFd)	*P* value	*F*(DFn, DFd)	*P* value	*F*(DFn, DFd)	*P* value
Two-way ANOVA of insoluble pTau normalized to insoluble human tau						
pT149	*F*(1, 14) = 15	*P* < 0.005	*F*(1, 14) = 0.001	*P* = 0.97	*F*(1, 14) = 0.01	*P* = 0.92
pT153	*F*(1, 14) = 3.8	*P* = 0.07	*F*(1, 14) = 1.0	*P* = 0.33	*F*(1, 14) = 0.16	*P* = 0.70
AT270	*F*(1, 14) = 7.73	*P* ≤ 0.05	*F*(1, 14) = 33.44	*P* < 0.0001	*F*(1, 14) = 0.66	*P* = 0.43
CP13	*F*(1, 14) = 0.25	*P* = 0.62	*F*(1, 14) = 1.59	*P* = 0.23	*F*(1, 14) = 4.47	*P* ≤ 0.05
pT205	*F*(1, 14) = 5.4	*P* ≤ 0.05	*F*(1, 14) = 1.9	*P* = 0.19	*F*(1, 14) = 0.01	*P* = 0.92
AT8	*F*(1, 14) = 24	*P* < 0.0005	*F*(1, 14) = 64	*P* < 0.0001	*F*(1, 14) = 8.5	*P* ≤ 0.05
pT212	*F*(1, 14) = 1.05	*P* = 0.32	*F*(1, 14) = 0.03	*P* = 0.87	*F*(1, 14) = 0.12	*P* = 0.74
pS214	*F*(1, 14) = 0.02	*P* = 0.90	*F*(1, 14) = 0.07	*P* = 0.79	*F*(1, 14) = 2.17	*P* = 0.16
AT100	*F*(1, 14) = 0.25	*P* = 0.63	*F*(1, 14) = 1.49	*P* = 0.24	*F*(1, 14) = 4.89	*P* = 0.04
PHF1	*F*(1, 14) = 0.76	*P* = 0.40	*F*(1, 14) = 11.63	*P* < 0.005	*F*(1, 14) = 4.14	*P* = 0.06
_TauP301L = 4 females, 5 males; LRRK2/TauP301L =5 females, 4 males_						
Two-way ANOVA of Tau Cortical Burden						
pT149	*F*(1, 14) = 4.5	*P* ≤ 0.05	*F*(1, 14) = 13	*P* < 0.005	*F*(1, 14) = 0.14	*P* = 0.71
pT153	*F*(1, 14) = 5.0	*P* ≤ 0.05	*F*(1, 14) = 14	*P* < 0.005	*F*(1, 14) = 0.53	*P* = 0.48
AT270	*F*(1, 14) = 0.22	*P* = 0.65	*F*(1, 14) = 0.24	*P* = 0.63	*F*(1, 14) = 0.32	*P* = 0.58
CP13	*F*(1, 14) = 4.6	*P* ≤ 0.05	*F*(1, 14) = 6.0	*P* ≤ 0.05	*F*(1, 14) = 0.17	*P* = 0.69
AT8	*F*(1, 14) = 8.5	*P* ≤ 0.05	*F*(1, 14) = 16	*P* < 0.005	*F*(1, 14) = 2.1	*P* = 0.17
MC1	*F*(1, 14) = 6.1	*P* ≤ 0.05	*F*(1, 14) = 8.8	*P* ≤ 0.05	*F*(1, 14) = 0.76	*P* = 0.40
_TauP301L = 5 females, 4 males; LRRK2/TauP301L = 5 females, 4 males_						

*Top* statistical results from a two-way ANOVA (genotype × sex) of the densitometry of phospho-specific tau antibodies normalized to the amount of human tau in the insoluble fraction for each mouse. *Bottom* Statistical results from a two-way ANOVA (genotype × sex) of cortical staining with phospho- and conformational-specific tau antibodies

### LRRK2/Tau_P301L_ mice have enhanced cortical tau pathology

**Fig. 6:**
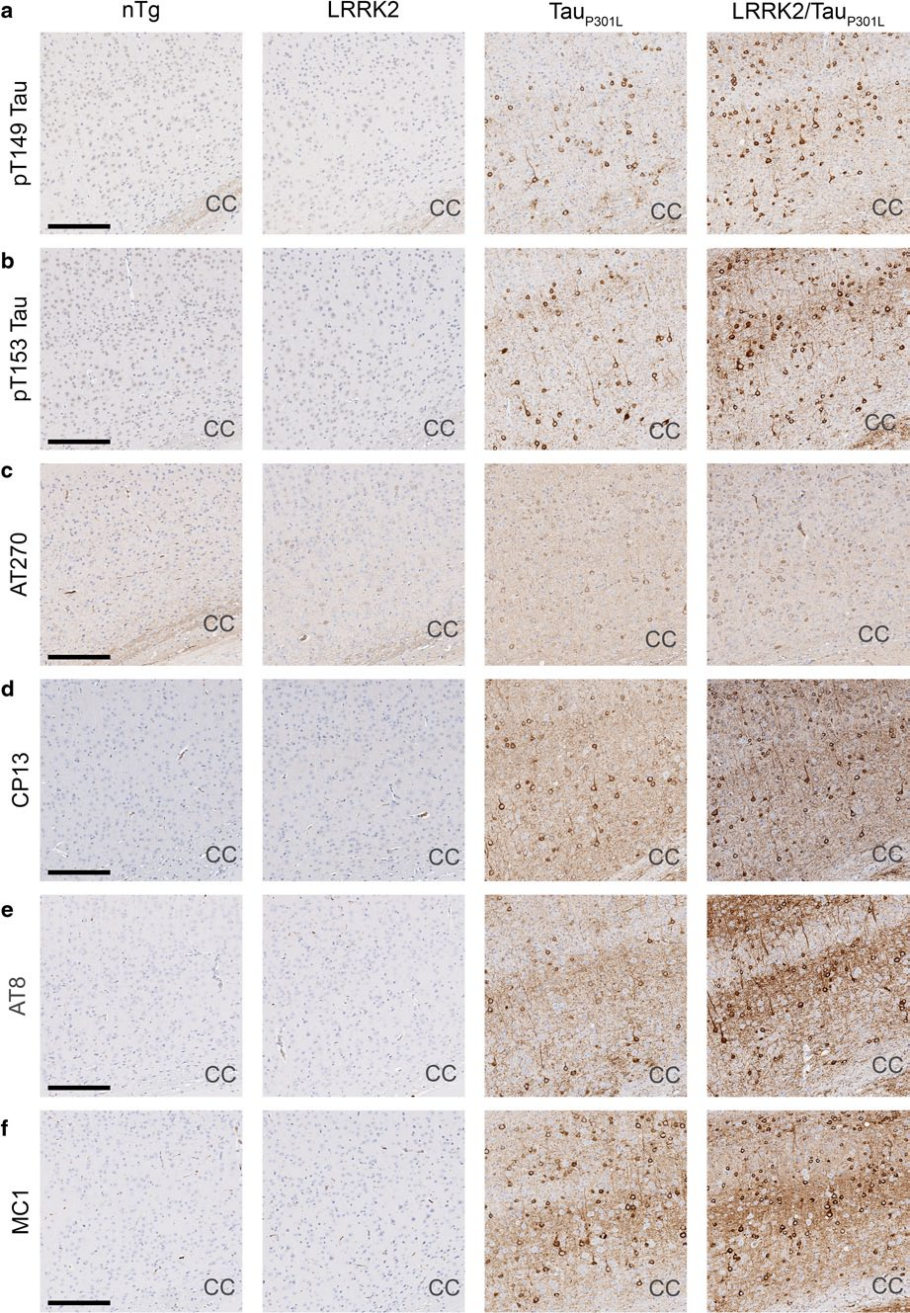
Cortical staining of phospho- and conformational-specific tau antibodies in female non-transgenic (nTg), LRRK2 only, Tau_P301L_ and LRRK2/Tau_P301L_ mice. **a**–**f** Representative images showing staining in the primary sensory cortex of female nTg, LRRK2, Tau_P301L_ and LRRK2/Tau_P301L_ mice with pT149 tau (**a**), pT153 tau (**b**), AT270 (pT181 tau) (**c**), CP13 (pS202) (**d**), AT8 (pS199/S202/T205 tau) (**e**), MC1 and (**f**) antibodies. *Scale bar* 200 μm. *CC* corpus callosum

**Fig. 7:**
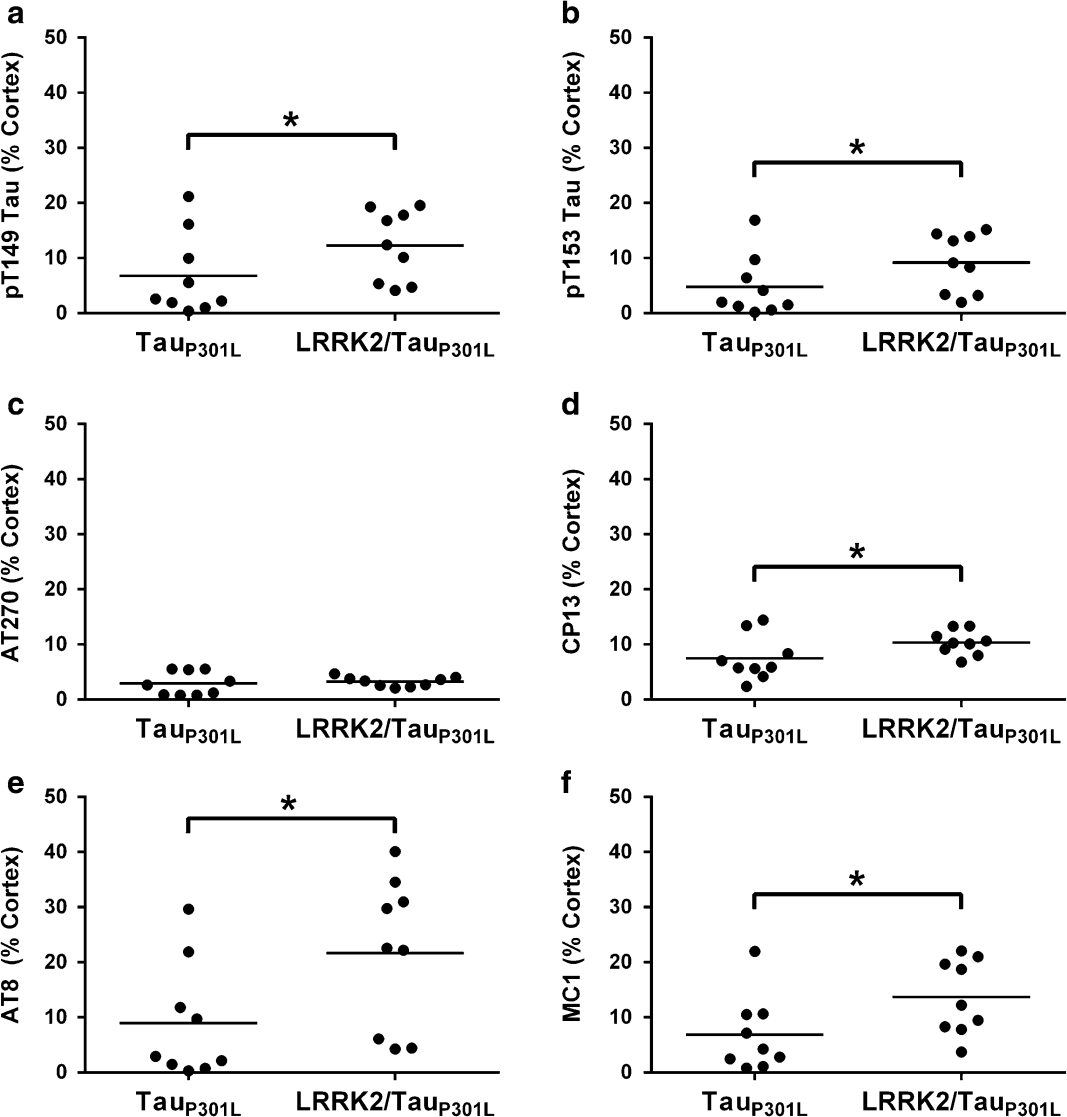
LRRK2/Tau_P301L_ have elevated levels of cortical tau pathology compared to Tau_P301L_ mice. **a**–**f** There are specific increases of tau phosphorylation at epitopes T149, T153, S202 (CP13), S199/S202/T205 (AT8), as well as tau with an abnormal conformation (MC1) in LRRK2/Tau_P301L_ mice versus Tau_P301L_ mice. No difference in phosphorylation of T181 (AT270) was observed when LRRK2/Tau_P301L_ mice were compared to Tau_P301L_ mice. *Each dot* represents an individual mouse with the mean indicated by the *black line*, *n* = 9 per cohort. *P ≤ 0.05 [two-way ANOVA (genotype × sex): main effect of genotype indicated]. Summary of two-way ANOVA found in Table 1

We sought to validate our biochemical studies with neuropathological analysis of tau burden using antibodies that detect specific phosphorylation or conformational tau epitopes. All antibodies identified prominent tau pathology, including neurofibrillary tangles, in the brains of Tau_P301L_ and LRRK2/Tau_P301L_ mice, which was absent in the LRRK2 only transgenic and non-transgenic (nTg) mice (Fig. 6). Tau pathology was distributed throughout the cortex and hippocampus of Tau_P301L_ and LRRK2/Tau_P301L_ mice; however, we focused our assessment on the cortex since neuronal loss within the hippocampus can confound accurate assessment of tau burden at this age. Using two-way ANOVA analysis, we observed significant increases in phosphorylated tau pathology in LRRK2/Tau_P301L_ mice with pT149, pT153, CP13, and AT8 immunostaining when compared to Tau_P301L_ mice (Figs. 6, 7; Table 1). Although the burden of tau pathology with these antibodies was also significantly influenced by sex, where females had greater burden than males, there was no interaction between the genotype and sex (Fig. 7; Table 1). In addition, we observed significant increases in conformationally-abnormal tau in LRRK2/Tau_P301L_ mice with MC1 immunostaining when compared to Tau_P301L_ mice (Figs. 6, 7; Table 1). Tau burden as assessed with MC1 was also significantly influenced by sex, but there was no interaction between genotype and sex (Table 1). No significant difference in tau burden was observed with AT270, irrespective of genotype or sex (Figs. 6, 7; Table 1).

## Discussion

In the current study, we combined in vitro, cell culture, and novel transgenic studies to demonstrate that tau is a substrate of LRRK2 and that this interaction promotes tauopathy. We found that recombinant WT and mutant LRRK2 directly phosphorylates tau in kinase assays. As shown previously for other LRRK2 substrates, G2019S LRRK2 yields the greatest levels of tau phosphorylation [9, 21, 31, 74]. Our subsequent in vitro studies used G2019S LRRK2 to obtain the highest levels of substrate phosphorylation, thereby reducing the chance that LRRK2-directed phosphorylation of tau would be inadvertently missed. Using MS analysis, we uncovered the tau epitopes that were potentially targeted by LRRK2 phosphorylation. To reduce false positives, MS analysis was performed in parallel with reactions utilizing KD LRRK2, and we further validated subsequent hits using LRRK2 kinase reactions coupled with site directed mutagenesis at identified sites of phosphorylation to block LRRK2 activity. Surprisingly, we identified tau T149 and T153 as a preferential target and a secondary target, respectively, of LRRK2-directed phosphorylation. Although much of our studies focused on mutant LRRK2, we sought to determine if tau could be a substrate of LRRK2 in vivo by generating novel transgenic mice, which expressed human WT LRRK2 and mutant tau. WT LRRK2 mice alone do not develop tau abnormalities and the tau abnormalities observed in mutant LRRK2 mice are modest [40, 41, 46]. For our LRRK2/Tau_P301L_ model, we combined a WT LRRK2 BAC line [46] with a Tau_P301L_ (rTg4510) model [65]. The rTg4510 model represents a well-characterized model, providing a “primed system” in which we could determine if human LRRK2 phosphorylates tau in vivo and if this could influence the development of tauopathy. We demonstrated that human WT LRRK2 expression in a mouse model of tauopathy enhances tau aggregation and tau hyperphosphorylation—critical features of human tauopathy.

Having identified T149 and T153 on tau as primary targets of direct G2019S LRRK2 phosphorylation in vitro, we then sought to determine the relevance of these sites to human tauopathy. One study has reported that T149 tau is phosphorylated by recombinant CK1δ and GSK-3β kinases [23]; however, phosphorylation of T149 has not been studied in vivo or associated with disease. Phosphorylation of T153 has been described in vitro and in cell culture [23, 29, 66] and one study shows that phospho-T153 (pT153) antibody labels neurofibrillary tangles in AD brain [2]. It is still unknown to what extent phosphorylation of T153 is associated with other tauopathies. We created antibodies specific for tau phosphorylated at T149 and at T153, respectively (see “Materials and methods”). We then confirmed their specificity in vitro and in cell culture and demonstrated the presence of these phospho-epitopes in neuronal and glial lesions of 3R tauopathies (PiD), 4R tauopathies (PSP) and 3R + 4R tauopathies (AD) and in G2019S-LRRK2 carriers (PD). There was also immunoreactivity in a subset of Lewy bodies, similar to the pattern we previously noted with other antibodies to phospho-tau [30]. T149 and T153 are largely unexplored tau epitopes, but it is of interest that they flank a rare variant in tau, A152T, that may be a risk factor for tauopathies such as PSP [7, 34, 36]. LRRK2 is not known to play a role in tauopathies beyond its involvement in PD, but it is possible that rare genetic variants in LRRK2, including those that confer risk to PD [16, 60, 62] or have yet to be uncovered, could play a role in tauopathies. The pT149 and pT153 immunostaining in these diverse cases of human tauopathy suggests that further studies on the role of LRRK2 in these disorders could be informative.

No changes to tau levels or phosphorylation were observed in the soluble fraction of LRRK2/Tau_P301L_ mice compared to Tau_P301L_ mice. Initial analysis of the insoluble fraction revealed increased phosphorylation of all epitopes examined in LRRK2/Tau_P301L_ mice. This was not surprising, however, as there was approximately three times more tau in the insoluble fraction of LRRK2/Tau_P301L_ mice. To account for the significant difference of insoluble tau in Tau_P301L_ only and LRRK2/Tau_P301L_ mice, it was necessary to adjust the phospho-tau levels to the total amount of insoluble tau present to fully assess if there were specific phosphorylation changes associated with LRRK2 overexpression. We found that co-expression of LRRK2 in Tau_P301L_ mice selectively increased insoluble tau phosphorylation at sites identified in vitro as being directly phosphorylated by LRRK2, T149 and T153, as well as the T205 and S199/S202/T205 epitopes. Interestingly, our in vitro results were performed in the context of soluble tau; whereas, the elevated phosphorylation in the mice in the presence of LRRK2 was only in insoluble tau. These data suggest that LRRK2-associated phosphorylation may be able to trigger the shift of soluble tau into the insoluble fraction. Davies et al. [13] have isolated a substantial amount of LRRK2 in the insoluble protein fraction using a Triton and SDS preparation and demonstrated that LRRK2 is not exclusively soluble in WT rodents or in humans. This could support a potential interaction of LRRK2 and tau in LRRK2/Tau_P301L_ mice as tau switches from its highly soluble (normal) state to the insoluble protein that is found in tauopathy. Alternatively, filamentous tau may be a better substrate for LRRK2 compared to the soluble tau. Neuropathological analysis revealed increased tau burden using antibodies for tau phosphorylated at T149, T153, S202, and pS199/S202/T205, largely replicating what we observed in our biochemical analysis. By both biochemical and histological analyses enhanced phosphorylation was detected by the AT8 antibody that recognizes a triple epitope (pS199/S202/S205). It is possible that these findings are due to increased phosphorylation at S202 or T205 that was observed with antibodies that specifically detect those singular epitopes.

Our in vitro and in vivo findings support that T149 tau is the primary target of direct LRRK2 phosphorylation and suggests that T153 may be a secondary target. Our MS studies identified T205 as a potential LRRK2 phosphorylation site which was not validated in our subsequent in vitro work utilizing synthetic peptides for this region; therefore, the increased phosphorylation of insoluble tau at T205 and the combined S199/S202/T205 epitope, recognized by the AT8 antibody, may indicate an indirect mechanism by which LRRK2 increases phosphorylation of these specific tau epitopes in vivo. Alternatively, phosphorylation of T205 may require unique modeling of secondary and tertiary structure, and therefore be a better substrate for LRRK2 in vivo than it is in vitro. In addition, microtubules or other co-factors may act as scaffold to bring tau and LRRK2 together, allowing LRRK2 to phosphorylate tau at additional epitopes [35]. LRRK2 might also directly phosphorylate other tau kinases and enhance their ability to target tau [49, 76], including AKT, GSK-3β, and members of the MAPK family, which have been implicated downstream of LRRK2 activity [4, 20, 42, 44, 53, 57]. In addition, LRRK2 phosphorylation of its main target epitopes (i.e., T149) may have the ability to enhance phosphorylation of additional epitopes by other tau kinases; such cooperation has been noted with other phospho-tau epitopes [45, 77]. Further experiments examining the interaction between LRRK2-associated tau phosphorylation and tau phosphorylation by known tau kinases are required to test this.

For proteins that are highly phosphorylated, such as tau, it is common to find redundancy of phosphorylation of a given residue by multiple kinases. For example, S202 tau has been shown to be phosphorylated by at least 8 different kinases, including CK1 (reviewed in [37]). CK1δ has also been shown to phosphorylate T149 tau in vitro [23] and interestingly, both CK1δ and LRRK2 have been shown to phosphorylate the disease-related α-synuclein protein at S129 [50, 57]. Given this, it would not be surprising if both kinases could phosphorylate tau at the same epitope. In *Drosophila*, LRRK2 has been proposed to increase tau phosphorylation at T212 in a GSK-3β dependent manner [42]. In our LRRK2/Tau_P301L_ mice, we did not detect increased phosphorylation of tau at T212 nor at S396/S404, a second epitope phosphorylated by GSK-3β [38], indicating that LRRK2 expression in Tau_P301L_ mice did not ubiquitously increase phosphorylation of tau epitopes targeted by GSK-3β (reviewed in [24]). Surprisingly, we observed a significant decrease in phosphorylation of insoluble tau at T181 in the LRRK2/Tau_P301L_ compared to Tau_P301L_ mice. Kawakami et al. [35] previously reported that LRRK2 did not promote phosphorylation of tau S199/S202/T205 in vitro and instead increased phosphorylation of T181 in cell culture and in vitro, a modification of tau that required the interaction with microtubules. Ujiie et al. [71] reported that LRRK2 modestly enhanced T181 phosphorylation in cell culture. The discrepancy between our findings and these reports may arise from differences between constructs or reaction design. Furthermore, the LRRK2/Tau_P301L_ mice express mutant tau, which has reduced tubulin binding [25, 27], potentially decreasing our ability to uncover tubulin-dependent LRRK2 phosphorylation of tau.

The evolution of toxic tau is a complex event, with no consensus on the biochemical switch from soluble to insoluble tau or functional to dysfunctional species. Our neuropathological analysis of LRRK2/Tau_P301L_ mice compared to Tau_P301L_ mice revealed elevated immunostaining with the MC1 antibody, which detects tau in an abnormal, disease-relevant conformation [73], agreeing with our biochemical findings of increased 64 kDa sarkosyl-insoluble tau. Although our in vitro results suggest that the increase in tauopathy in the LRRK2/Tau_P301L_ mice is likely derived from the kinase function of LRRK2, it is possible that in vivo LRRK2 also promotes the aggregation of tau into the insoluble fraction by indirect cellular mechanisms. Other functions such as its regulation of autophagy have been assigned to LRRK2, which may contribute to our in vivo observations. In addition, some findings suggest that LRRK2-mediated tau phosphorylation can inhibit microtubule binding [35], which could also promote tau aggregation. Further studies will be required to determine the relative contribution of these alternative mechanisms on tau aggregation.

In some cases, we detected an influence of sex as well as genotype on neuropathological and biochemical outcomes in our mouse studies when assessed by two-way ANOVA. Neither transgenic tau nor LRRK2 expression was influenced by sex; therefore, these differences are not easily explained, but they are interesting and could be physiologically important. Curiously, the age-associated cumulative incidence of LRRK2 G2019S PD in Tunisia is gender specific—the median age of onset of female carriers being 5 years younger (in preparation, Matthew J. Farrer). In our lab, we have observed a non-significant trend that female rTg4510 mice have steeper exponential phase of tau pathology than male rTg4510 mice between ~4 and 6 months of age (personal communication, Jada Lewis). This inherent difference may be amplified by LRRK2 influence on tau pathology.

Our data, in aggregate, demonstrate that LRRK2 directly phosphorylates tau at T149 and T153 in vitro and the ability of LRRK2 to phosphorylate tau at these sites may underlie its ability to promote tauopathy in our novel mouse model. Our current in vivo studies are the first of their kind and provide compelling evidence that LRRK2 and tau interact in a disease-relevant manner. Further, the presence of phosphorylation at tau T149 and T153 in a variety of tau pathologies suggests that LRRK2 genetic studies in human tauopathies may be warranted.

## Electronic supplementary material

Below is the link to the electronic supplementary material.

**Supplemental Figure 1** Full-length G2019S LRRK2 phosphorylates multiple isoforms of tau with and without FTDP-17*t* mutations. Recombinant full-length GST-G2019S LRRK2 (1–2,527) phosphorylates tau, regardless of tau isoform or mutation as shown by autoradiography. Myelin basic protein (MBP) was used as a positive control for LRRK2 kinase activity. Coomassie blue shows similar loading of tau constructs. Tau mutation abbreviations: EV = E342V; PL = P301L; PS = P301S; RW = R406W. * indicates endogenous GST from HEK 293T cells purified with GST-LRRK2 (1–2,527).

**Supplemental Figure 2** Breeding scheme ensures LRRK2/Tau_P301L_, Tau_P301L_ and control mice are on the same strain background. (**Top**) BAC WT LRRK2 mice on the FVB background strain were crossed with Tau-Responder mice, also on the FVB background strain, for one generation. Bigenic LRRK2.Tau_-_Responder mice (**middle**) on an FVB background strain result from the aforementioned crossbreeding. The tau responder is not induced in these bigenic animals due to the absence of the tTA transgene. Bigenic LRRK2.Tau-Responder mice were then crossed with tTa effector mice on the 129S background strain for one generation to obtain the F1 triple transgenic LRRK2/Tau_P301L_, Tau_P301L_ and control mice (**bottom**) on a 50 % FVB/50 % 129S background strain. This maintained the same background strain as the original rTg4510 mouse model of tauopathy. The mice that are boxed and crossed out indicate those mice that were not used in this study.

**Supplemental Figure 3** Phosphorylation of soluble tau is unchanged by LRRK2 expression in Tau_P301L_ mice. (**a-b**) Western blot analyses of the soluble fractions of whole brain lysates from Tau_P301L_ only and LRRK2/Tau_P301L_ mice. (**a**) Representative immunoblots and (**b**) densitometric quantification of the soluble fraction of LRRK2/Tau_P301L_ and Tau_P301L_ mice probed with antibodies specific for phosphorylation of tau at T149, T153, T181 (AT270), S202 (CP13), T205, S199/S202/T205 (AT8), T212, S214, T212/S214 (AT100), and S396/S404 (PHF1). Western blots were normalized with GAPDH. Graphs in (**b**) only quantify the ~ 55 kDa species of tau which represents the normal molecular weight. No differences were detected in levels of phosphorylation of soluble tau between LRRK2/Tau_P301L_ mice and Tau_P301L_ mice. Each dot represents an individual mouse with the mean indicated by the black line, *n* = 9 per cohort.

**Supplemental Figure 4** Both female and male LRRK2/Tau_P301L_ mice have increased tau aggregation compared to same sex Tau_P301L_ only mice. (**a**) (**Top**) Representative immunoblot of female Tau_P301L_ and LRRK2/Tau_P301L_ mice and (**bottom**) densitometric quantification. (**b**) (**Top**) Representative immunoblot of male Tau_P301L_ and LRRK2/Tau_P301L_ mice and (**bottom**) densitometric quantification. Each dot represents an individual mouse with the mean indicated by the black line, *n* = 4–5 per cohort (females = red; males = blue). * *P* ≤ 0.05, ***P < 0.001 (Student’s t test, unpaired, two-tailed).

**Supplemental Figure 5** Aggregation of hyperphosphorylated insoluble tau is increased in LRRK2/Tau_P301L_ versus Tau_P301L_ mice. (**a-k**) Western blot analyses of the sarkosyl-insoluble fractions of whole brain lysates from Tau_P301L_ only and LRRK2/Tau_P301L_ mice. (**a**) Representative immunoblot probed with an antibody (E1) that recognizes human tau, regardless of phosphorylation state, shows more aggregated (insoluble) human tau in LRRK2/Tau_P301L_ compared to Tau_P301L_. (**b-k**) Representative western blots of sarkosyl-insoluble fraction probed with antibodies specific for phosphorylation of tau at T149 (**b**), T153 (**c**), T181 (AT270) (**d**), S202 (CP13) (**e**), T205 (**f**), S199/S202/T205 (AT8) (**g**), T212 (**h**), S214 (**i**), T212/S214 (AT100) (**j**), and S396/S404 (PHF1) (**k**) show elevated levels of tau phosphorylated at each epitope in LRRK2/Tau_P301L_ mice compared to Tau_P301L_ mice.

**Supplemental Figure 6** Phosphorylated, insoluble tau is generally increased in LRRK2/Tau_P301L_ versus Tau_P301L_ mice. (**a-j**) Densitometry of the western blots (shown in **Supplemental Fig. 5**) for phosphorylation of tau at T149 (**a**), T153 (**b**), T181 (AT270) (**c**), S202 (CP13) (**d**), T205 (**e**), S199/S202/T205 (AT8) (**f**), T212 (**g**), S214 (**h**), T212/S214 (AT100) (**i**), and S396/S404 (PHF1) (**j**) within the sarkosyl-insoluble fraction indicates that there is a general increase in phosphorylation of this fraction in LRRK2/Tau_P301L_ compared to Tau_P301L_ mice before they have been normalized for total amount of aggregated insoluble tau. Each dot represents an individual mouse with the mean indicated by the black line, *n* = 9 per cohort. **P < 0.01, ***P < 0.001, ****P < 0.0001 [two-way ANOVA (genotype x sex): main effect of genotype indicated].

Supplementary material 1 (PDF 2290 kb)
