# Supplementary material for: LRRK2 phosphorylates novel tau epitopes and promotes tauopathy
Source: Acta Neuropathol. 2013 Oct 11;126(6):809–27. doi: 10.1007/s00401-013-1188-4 (PMC3830748; doi:10.1007/s00401-013-1188-4)
Supplement: Supplementary file 1 — Supplementary material 1 (PDF 2290 kb) [file 401_2013_1188_MOESM1_ESM.pdf]

LRRK2 Phosphorylates Novel Tau Epitopes and Promotes Tauopathy

Rachel M. Bailey[1, 2], Jason P. Covy[3, 4], Heather L. Melrose[2], Linda Rousseau[2], Ruth Watkinson[2], Joshua Knight[1, 2], Sarah Miles[2], Matthew J. Farrer[2, 5], Dennis W. Dickson[2], Benoit I. Giasson[1, 3*], and Jada Lewis[1, 2*]

[1] Center for Translational Research in Neurodegenerative Disease and Department of Neuroscience, University of Florida, Gainesville, FL 32610, USA., [2] Department of Neuroscience, Mayo Clinic, Jacksonville, FL 32224, USA., [3] Department of Pharmacology, University of Pennsylvania School of Medicine, Philadelphia, PA 19104, USA., [4] Department of Molecular & Cellular Physiology, Stanford University, Stanford, CA, 94305, [5] Department of Medical Genetics, University of British Columbia, Vancouver, British Columbia V6T 2B5, Canada.

Abbreviated title: LRRK2 phosphorylates tau

***Address correspondence to:**

Dr. Jada Lewis, Department of Neuroscience, Center for Translational Research in Neurodegenerative Disease, University of Florida, 1275 Center Drive, BMS Building J-487, PO Box 100159, Gainesville, FL32610-0244. Tel: 352-273-9666; E-mail: jada.lewis@ufl.edu.

Dr. Benoit Giasson, Department of Neuroscience, Center for Translational Research in Neurodegenerative Disease, University of Florida, 1275 Center Drive, BMS Building J-497, PO Box 100159, Gainesville, FL32610-0244. Tel: 352-273-9363; E-mail: bgiasson@ufl.edu.

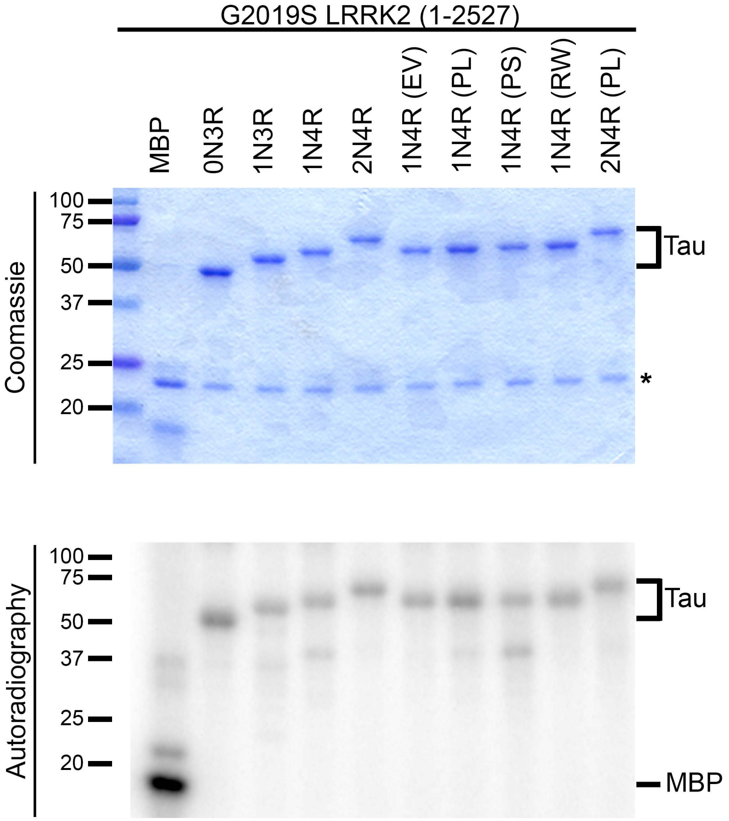

Bailey et al Supplemental Fig. 1

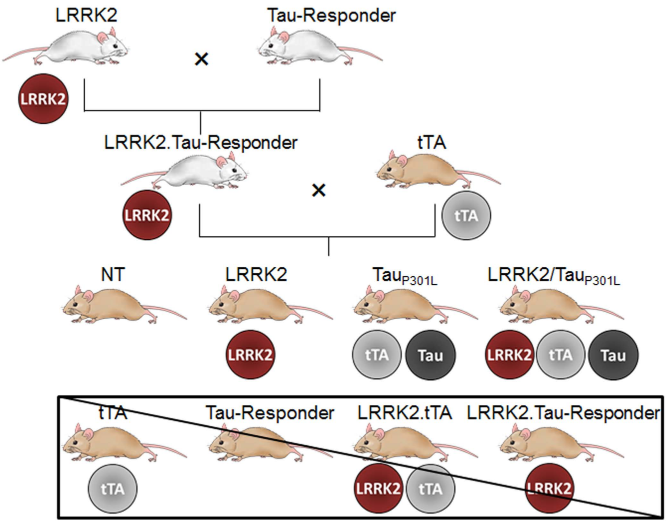

Bailey et al Supplemental Fig. 2

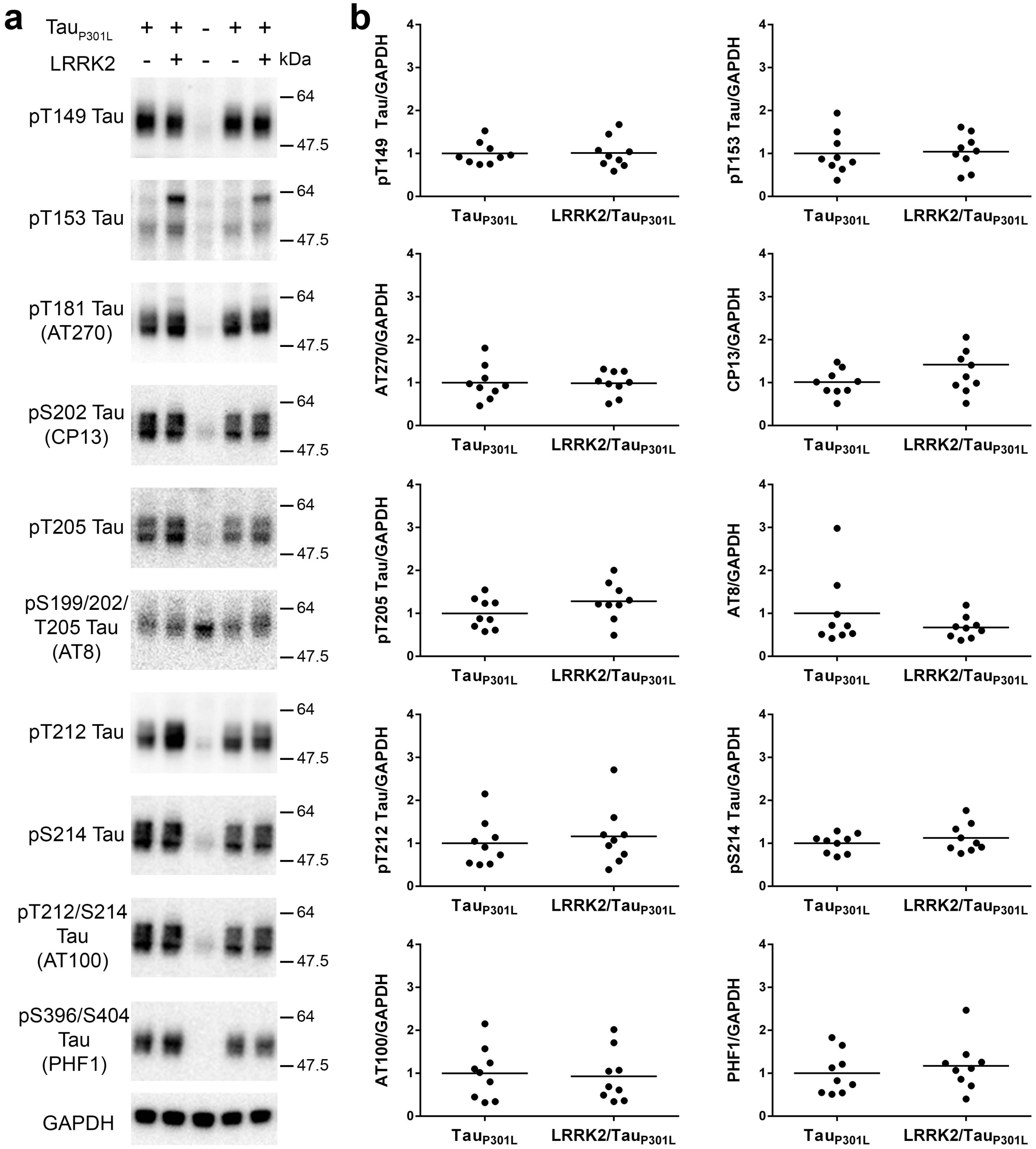

Bailey et al Supplemental Fig. 3

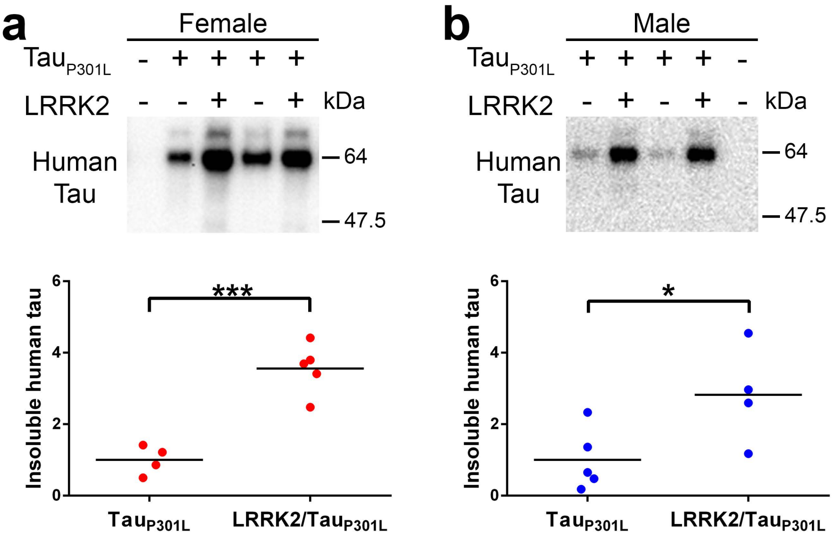

Bailey et al Supplemental Fig. 4

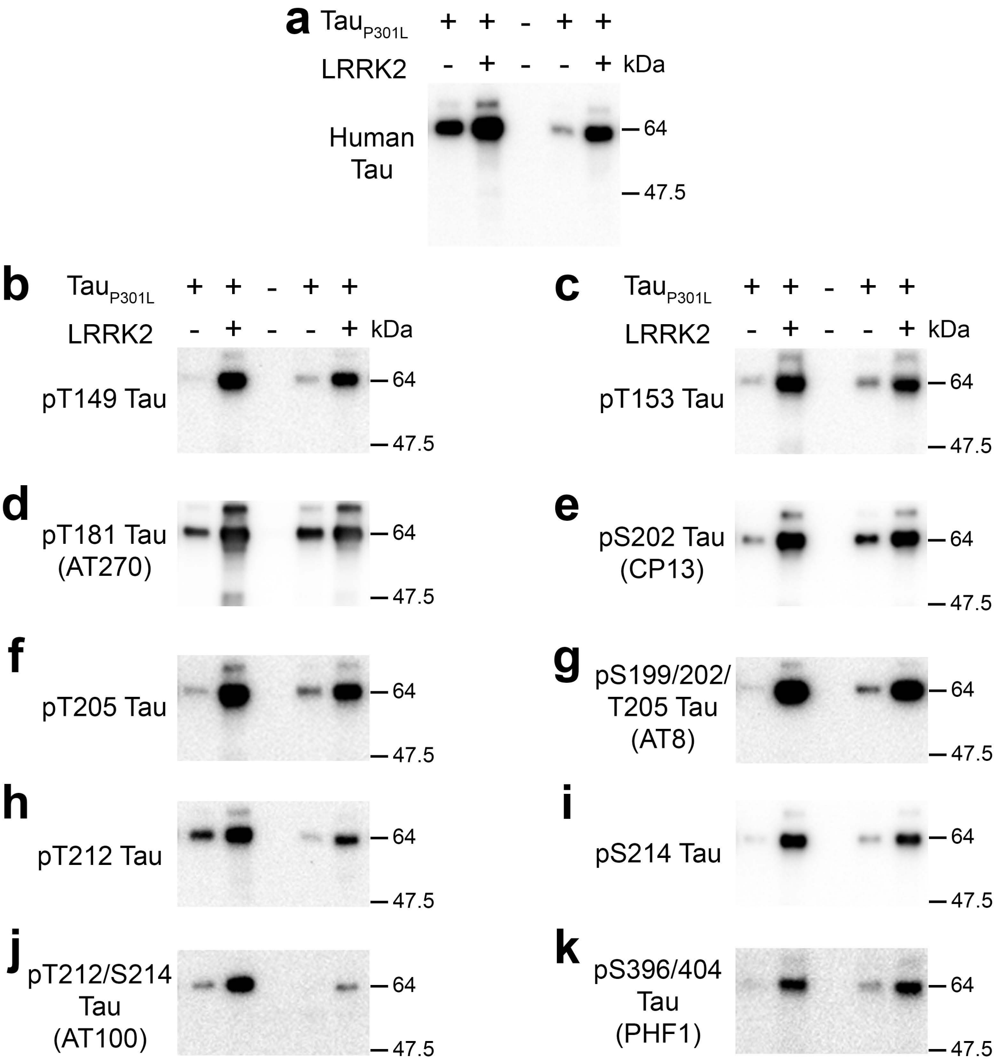

Bailey et al Supplemental Fig. 5

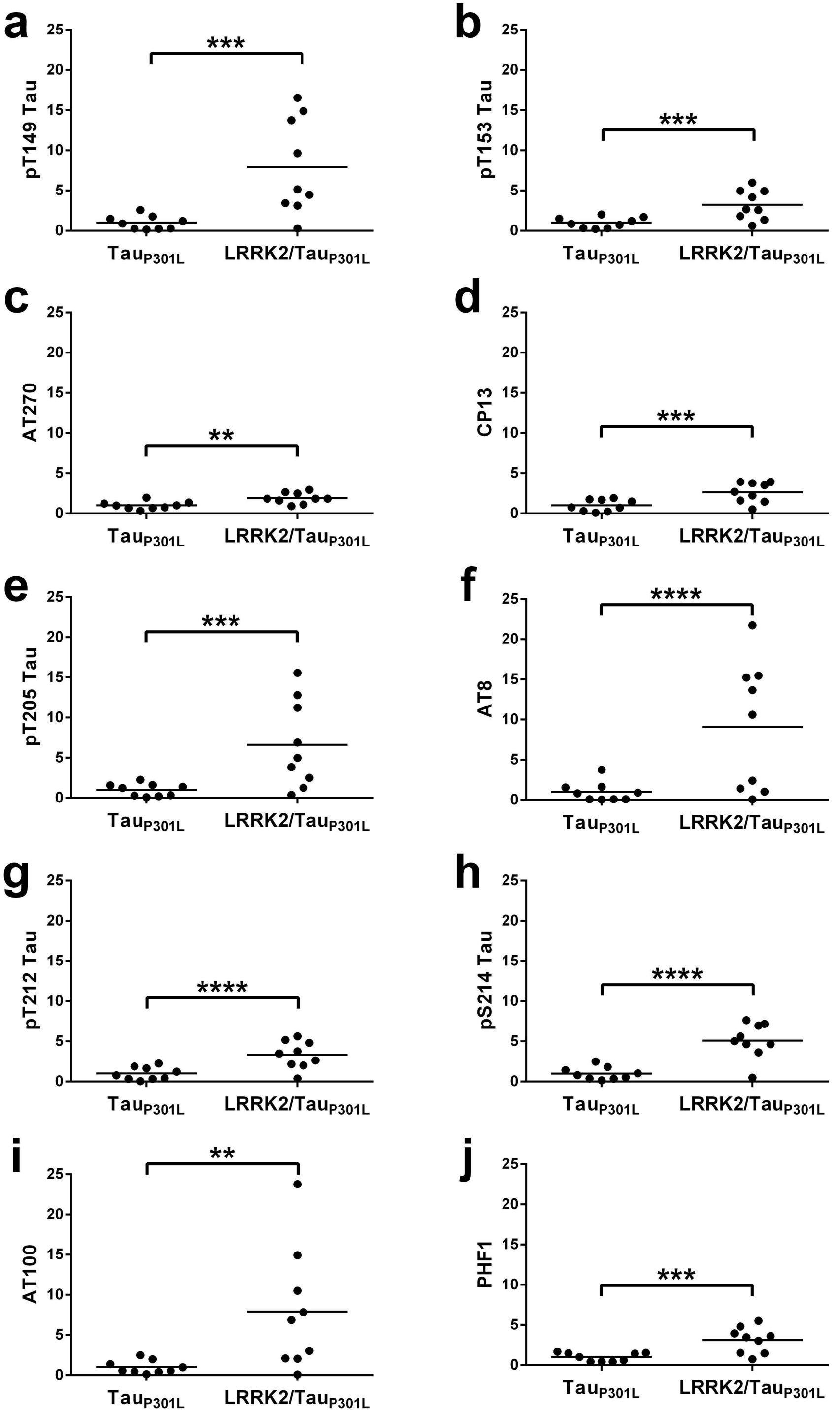

Bailey et al Supplemental Fig. 6

**Supplemental Table 1A.** Tau residues phosphorylated *in vitro* by LRRK2	
**Residue**	**% Phosphorylation**
T30	5.3
T135	0.5
**T149**	**16.8**
**T153**	**13.2**
**T169**	**47.1**
T175	8.7
T181	6.1
**T205**	**23.3**
S210	9.1
T212	3.8
T217	11.1
**T231**	**12.8**
S237	2.3
T245	9.4
S258	3.4
**T263**	**15.8**
S324	8.0
S356	2.2
T361	2.9
T373	1.5
T377	11.1
T386	3.7
Y394	3.6
T403	5.0
S435	2.0

**Supplemental Table 1B.** Tau residues potentially phosphorylated *in vitro* by LRRK2
**Residue**
T30
T17
S184
S185
S208
T220
S238
S262
S352
S413

**Supplemental Table 2.** Synthetic tau peptide list	
**Peptide**	**Amino Acid Coverage**
Tau-A	KKAKGADGKTKIATPRGAAPPGQK
Tau-B	REPKKVAVVRTPPKSPSSAKSRL
Tau-C	QKGQANATRIPAKTPPAPKTPPSSG
Tau-D	SGYSSPGSPGTPGSRSRTPSLPT
Tau-E	SSAKSRLQTAPVPMPDLK
Tau-F	DLKNVKSKIGSTENLKHQ
Tau-G	GQVEVKSEKLDFK
Tau-H	FKDRVQSKIGSLDN
Tau-I	DRVQSKIGSLDNITHVPGGGNKKIE
Tau-J	QSKIGSLDNITHVPGGG

Supplemental Table 2 Synthetic tau peptide sequences. Red = residues that were identified by mass spectrometry as LRRK2 phosphorylation targets.
